# A Unique Case of Small Bowel Internal Hernia and Volvulus Caused by a Hamartoma

**DOI:** 10.1055/s-0041-1733832

**Published:** 2021-08-17

**Authors:** Charif Khaled, Michel Akl, Toufic Moussallem

**Affiliations:** 1 Department of General Surgery, Faculty of Medical Sciences, Lebanese University, Beirut, Lebanon; 2 Faculty of Medical Sciences, Lebanese University, Beirut, Lebanon; 3 Department of General Surgery, General and Laparoscopic Surgeon, Sacred Heart Hospital, Beirut, Lebanon

**Keywords:** internal hernia, small bowel, volvulus, closed-loop obstruction, hamartoma, neoplasm

## Abstract

This study depicts the case of a young female presenting with intestinal obstruction. Surgery uncovered a small bowel hamartoma that has caused a transmesenteric internal hernia and volvulus. As far as we know, this is unheard of before, as all three mentioned entities are very rare. The study also covers a literature review of cases of internal hernia with volvulus and stresses over the need for urgent diagnosis and management.

## Clinical Case

Our case revolves around a previously healthy 28-year-old female who presented to the emergency department for acute onset of severe abdominal pain. She described the pain as being knife-life and diffuse, radiating to the back bilaterally. The patient also complained of nausea and several episodes of vomiting. She noted no changes in bowel habits. Finally, the patient denied any consumption of suspicious foods or drinks in the past few days.

Her vitals were normal upon presentation. The review of system did not add any other information. Physical examination was significant for a mildly distended abdomen and palpation revealed it to be soft but diffusely tender.

Withdrawn laboratories included complete blood count with differential, chem6, and liver and pancreatic enzymes. They were significant for leukocytosis at 19,000 cells/μL (4,000–10,500 cell/μL) with 97% (40–74%) neutrophils and microcytic anemia with a hemoglobin of 10.1 g/dL (12–14 g/dL) and mean corpuscular volume of 78 fL (80–100 fL). Due to the severity of the pain and high white blood count, a computed tomography (CT) scan of the abdomen and pelvis with intravenous contrast was ordered.

**Fig. 1:**
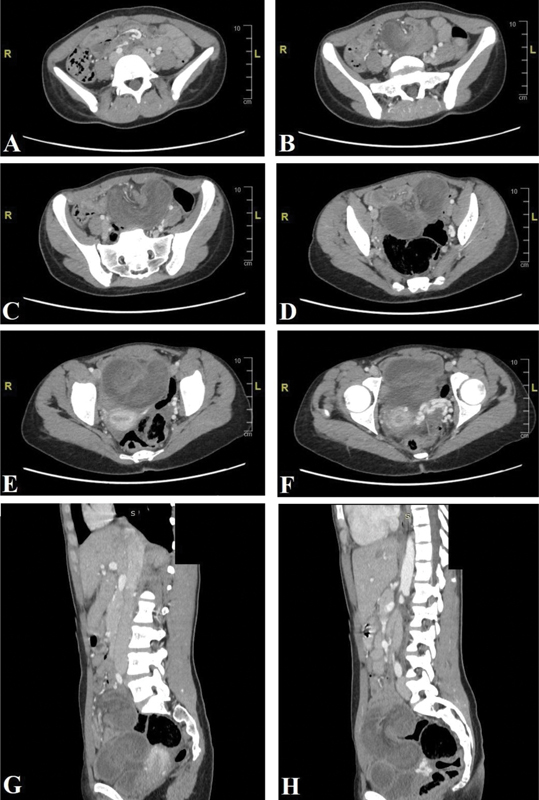
Computed tomography scan of the abdomen and pelvis + intravenous contrast.

The CT showed ( Fig. 1 ) a fluid-filled and distended small bowel segment (4 cm) with collapsed proximal and distal ends. This segment also shows wall enhancement, suggesting bowel suffering. Also noted is a “Whirlpool sign” of the adjacent mesentery; all these findings suggesting small bowel volvulus with a closed-loop obstruction. Finally, a small fluid collection was found in the pelvis.

**Fig. 2:**
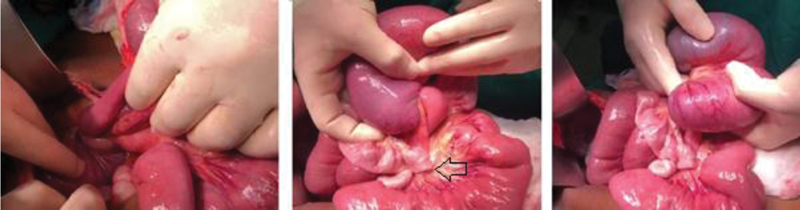
Intraoperative image of the bowel. Images show volvulus of an ischemic segment with flat proximal and distal ends.

After discovering these findings, the patient was scheduled for an urgent surgical exploration. Aggressive hydration was given preoperatively, written consent was taken and the patient was transferred to the operating room. A midline laparotomy was done. Upon examination of the bowels, an ischemic and distend segment of the terminal ileum was found herniated through the mesentery and rotated at 360 degrees ( Fig. 2 ). Hernia reduction and untwisting of the bowel was done. Upon palpation, a small tumor was sensed in the ischemic segment around which most likely the herniation had started and led to the volvulus. The affected segment of small bowel was necrotic and so it was resected. Latero-lateral mechanical anastomosis was then done. Running of the bowel did not reveal any other lesions and abdominal exploration did not show any gross abnormalities.

The patient's postoperative period was uneventful. Her Foley and nasogastric tube were removed on days 2 and 3, following which per os feeding was progressively started. She was discharged on day 6 postoperatively. On pathology, the lesion was found to be a hamartomatous polyp with associated enteritis cystica profunda. Immunostaining was positive for CDX2 but negative for CK7, suggesting the possibility of Peutz–Jeghers syndrome. Sadly, no additional tests could be done as the patient refused for financial reasons.

## Introduction

Internal hernia of the small bowel is a very rare entity. It can be challenging to diagnose and can rapidly lead to serious complications like volvulus, obstruction, and even bowel ischemia and gangrene. Rapid detection and treatment are crucial as they can save the patient's life.

## Discussion

Internal hernia is a unique entity with an incidence of 0.2 to 0.9%. The male-to-female ratio is 3:2. 1 Welch identified several types of internal hernia. By order of frequency, they are paraduodenal hernia (right and left), pericecal hernia, foramen of Winslow hernia, transmesenteric hernia, intersigmoid hernia, supravesical and pelvic hernia, and transomental hernia. 2 3 These were grouped by Doishita et al 4 into three main groups according to types of hernia orifices; normal foramen, unusual peritoneal fossa or recess into retroperitoneum, and abnormal opening in a mesentery or peritoneal ligament.

Our case is of the transmesenteric type and is relatively uncommon, with an incidence rate of 8% of all internal hernias. 3 However, transmesenteric hernias are on the rise due to Roux-en-Y anastomosis reconstruction. The small bowel is the most commonly herniated. 4 Nevertheless, some rare cases reported colon herniation. 1

Causes of internal hernia in adults are various but most commonly encompass previous gastrointestinal surgery, abdominal trauma, intraperitoneal inflammation, or neoplasm. 1 In our case, it is suspected that the hamartomes polyp had caused the small bowel traction and herniation. Hamartomas and small bowel neoplasms have been reported to cause intussusception. 5 Note that hamartomas usually present in the setting of Peutz–Jeghers syndrome. This syndrome is characterized by the development of noncancerous polyps in the gastrointestinal tract, mucocutaneous-pigmented macules, and increased lifetime risk of certain cancers. 6 But as far as we know, there are no reported cases of internal hernia caused by a hamartomatous lesion.

The clinical presentation can be deceiving. Internal hernia can be asymptomatic for years or present with nonspecific abdominal pain of distention. Note that, internal hernia accounts for 4% of acute intestinal obscuration. 4 When obstruction occurs, patients will present with acute onset of severe abdominal pain, abdominal distention, severe nausea and vomiting, constipation, and obstipation. 4 7 So, symptomatology mainly depends on the extent of complications. Presentation can be mild in noncomplicated cases and can be severe in cases of volvulus, strangulation, and ischemia, which occurs in 33% of cases 8 as our case. The mechanism of volvulus is explained by the lack of capsulation of the hernia, which allows larger loops to herniate and twist. 9

Imaging is the tool to accurately determine the diagnosis. Simple X-ray can show signs of intestinal obstruction or stacking of bowels in a specific quadrant of the abdomen. 8 Multidetector computed tomography scan is the gold standard imaging modality. Its specificity and sensitivity are 76 and 63%, respectively, with an accuracy of 77% for trans-mesenteric hernias. 1 Unless there is a contraindication, intravenous contrast is preferred as it allows visualization of blood flow to the involved intestine and helps in the assessment of the severity of small bowel strangulation, in comparison to nonenhanced CT which may show hyperattenuation of bowel wall, reflecting hemorrhagic congestion. As for oral contrast, water-soluble contrast can help identify the site and degree of obstruction, 4 but it can be very uncomfortable for the patient with complete or closed-loop obstruction and can lead to aspiration.

Diagnosis is not always reached via imaging. Following the acute and severe presentation, most patients are rushed for surgical exploration. Urgent surgical management is crucial for early accurate diagnosis and to prevent bowel ischemia and necrosis. Reduction of hernia/volvulus is the first step to be done. After that, lavage of ischemic bowel and inspection for nonviable segments should be done, and necrotic segments should be resected. Intestinal anastomosis cannot be always achieved and carries a high risk of leak and fistula, especially in severely distended bowel, but is the preferred option to stoma creation. 10 Anastomosis can be done manually or mechanically. The hernia orifice should be closed with nonabsorbable sutures to prevent recurrence. Despite recurrence risk, the foramen of Winslow is an exception and is not routinely closed to prevent complications such as portal venous thrombosis. 4

Open versus laparoscopic surgery is a topic of debate for every surgical case. Nevertheless, Jeong et al 11 reported shorter hospital stay, fewer complications, faster return to normal life, and better cosmetic results following laparoscopic surgery.

## Conclusion

The diagnosis of internal hernia is not always easy and so it should be kept in mind in front of every acute abdomen. Rapid diagnosis and management can prevent bowel necrosis and patient morbidity and even mortality.
